# Response of Plankton Community Under High-Nitrogen Ecological Replenishment: Niche Differentiation and Species Interactions

**DOI:** 10.3390/biology15201814

**Published:** 2026-10-11

**Authors:** Caili Du, Tao Wu, Zhenjun Tian, Jingxuan Wang, Xianzhe Zhang, Zhaozhi Liu, Jianglong Cui, Shengwang Gao, Lieyu Zhang

**Affiliations:** 1 State Key Laboratory of Environmental Criteria and Risk Assessment, Chinese Research Academy of Environmental Sciences, Beijing 100012, China; 2 Department of Environmental Science and Engineering, Fudan University, Shanghai 200433, China

**Keywords:** ecological water replenishment, high nitrogen, Lake Ulansuhai, plankton, niche, network structure

## Abstract

Ecological water replenishment is crucial for alleviating ecological degradation of lakes in cold and arid areas. However, especially if high-nitrogen water is used as a water source for replenishment, the water volume in lakes effectively increases, resulting in new environmental pressures, posing potential threats to aquatic ecosystems. Clarifying the response characteristics of plankton communities to high nitrogen replenishment is crucial for lake management. Under high nitrogen replenishment conditions, we focused on the ecological niche characteristics (including niche width and niche overlap), interspecies relationships and interaction networks of dominant phytoplankton and zooplankton species in Ulansuhai Lake in Inner Mongolia, with the aim of exploring the potential associations and response patterns of the plankton community. The results indicated that under high nitrogen replenishment conditions, the structure of plankton communities generally remained stable, with no significant fluctuations in species composition, density, biomass, or diversity indices. The niche width values of the dominant species were mostly above 0.6, suggesting that these species adopted niche-broadening strategies to enhance their environmental adaptability. Within the phytoplankton community, species exhibited a significant positive correlation, reflecting strong co-occurrence and niche differentiation. In contrast, interspecific relationships among zooplankton were not significant, reflecting asynchronous dynamics and different developmental trends under fluctuating environments. Co-occurrence network analysis further indicates that both phytoplankton and zooplankton form complex networks characterized by a high degree of modularity. Nevertheless, the phytoplankton and zooplankton networks in 2024 exhibited a reduction in size, an increase in negative cohesion (indicating heightened competition), and a decline in network robustness compared to 2023. The findings of this study provide a theoretical basis for the scientific implementation of ecological water replenishment and ecosystem management in cold and arid lakes under high-nitrogen conditions.

## Simple Summary

Ecological water replenishment facilitates the restoration of lakes in cold and arid regions, but high-nitrogen water may also impose additional stress on aquatic ecosystems. This study examined how phytoplankton and zooplankton communities in Ulansuhai Lake responded to high-nitrogen water replenishment. Overall, the plankton communities remained relatively stable, although their ecological networks became smaller, more competitive, and less robust over time. These findings provide useful information for improving ecological water replenishment and lake management.

## 1. Introduction

Lakes, as vital components of ecosystems, play a crucial role in maintaining the global ecological balance. However, in recent years, owing to the excessive exploitation of water resources, encroachment on lake ecosystems, and intensified anthropogenic activities such as industrialization, lakes worldwide have faced a series of severe challenges, including shrinking surface areas, declining water storage capacity, deteriorating water quality, and degradation of ecological functions [1,2,3]. More than 75% of enclosed water bodies worldwide (such as lakes and reservoirs) exhibit varying degrees of eutrophication and water quality deterioration, which may lead to water resource shortages and supply crises [4]. Against this backdrop, effectively improving and ensuring the sustainable development of lake ecosystems has become a significant global environmental concern.

Ecological water replenishment is an effective restoration measure to improve lake water quality and alleviate eutrophication [5,6,7,8]. Long-term monitoring data indicate that ecological water replenishment has significantly improved the eutrophic state of lakes, reducing them from a moderately eutrophicated state to a mildly eutrophicated state [9]. Notably, the composition and dynamic of biological communities are core drivers for maintaining freshwater ecosystem functions, and their diversity and stability collectively determine the environmental adaptability and disturbance resistance of the ecosystem [10]. However, in the context of ecological water replenishment, particularly exogenous input of high-nitrogen water, the original nutrient structure may be altered, thereby reshaping community structure through pathways that promote the spread of pollution-tolerant species and inhibit the growth of submerged plants. Interspecific ecological niches provide a crucial perspective for understanding community ecological characteristics, enabling the analysis of interactions between species and their environments, which in turn leads to the formation of unique environmental niches. This associative characteristic directly influences the community structure, function, and succession direction [11,12,13,14,15]. Systematically analyzing the changes in interspecific relationships under the influence of ecological water replenishment holds significant theoretical value for revealing the response mechanisms of lake ecosystems [16].

Lake Ulansuhai is a fluvial relic lake formed by a course change in the Yellow River. As one of the eight major freshwater lakes in China, its ecological functions are critically important. However, since the 1990s, owing to factors such as reduced natural water replenishment and cumulative industrial and agricultural pollution, the ecological functions of the lake have been severely degraded, with the water quality continuously deteriorating. To effectively improve the ecological environment of the lake, an ecological water replenishment project has been in place since 2012, primarily utilizing water resources during the Yellow River ice flood period and irrigation intervals to accelerate water exchange and alleviate eutrophication [9]. However, the high nitrogen content (>2.5 mg/L) in the inflow water of the Yellow River not only altered the lake’s original nutrient structure but also affected the composition of plankton communities and interspecies interactions. Therefore, this study aimed to investigate the potential associations and response patterns of plankton communities in Ulansuhai Lake under high-nitrogen ecological water replenishment. The specific objectives included (1) analyzing the structural response characteristics of lake plankton communities under high-nitrogen replenishment conditions, (2) evaluating the effects of high-nitrogen replenishment on the width and overlap of biological communities, and (3) revealing the changes in community interaction networks under high-nitrogen replenishment. Through a multidimensional analysis (including community structure, niche characteristics, and interaction networks), which systematically explored the structural features of plankton communities in lake ecosystems under high-nitrogen conditions, as well as providing a theoretical basis and scientific support for the ecological restoration, optimizing ecological water replenishment strategies, maintaining biodiversity, and preserving ecosystem functions of Ulansuhai Lake and other similar lakes.

## 2. Materials and Methods

### 2.1. Study Area and Sampling

**Figure 1:**
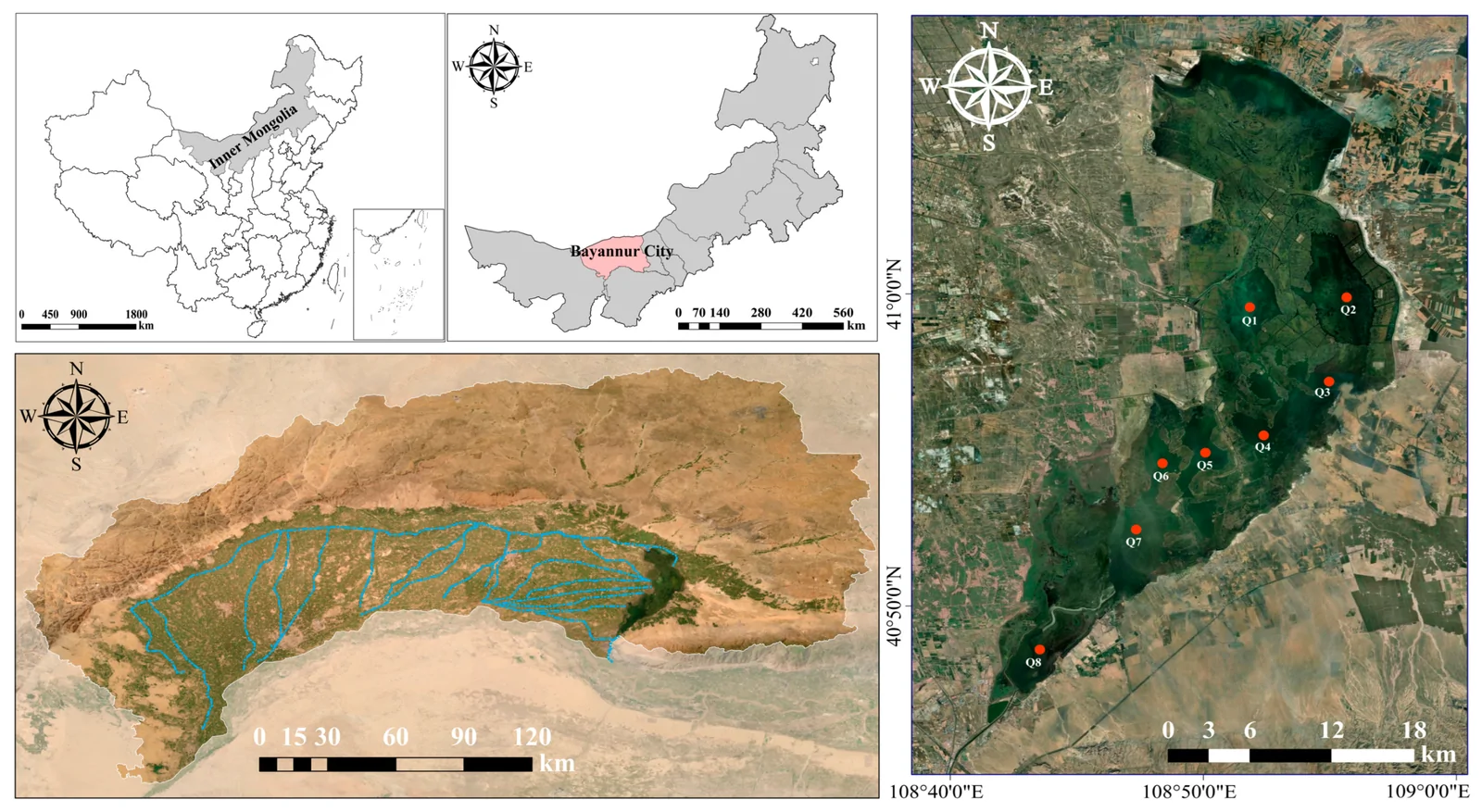
The distribution of sampling sites.

Ulansuhai Lake (40°36′–41°7′ N, 108°40′–109° E) is situated in the Hetao Plain of northwestern China. It is a large, low-lying lake with a water surface area of approximately 293 km^2^ (Figure 1) [17]. Located in a high-latitude, cold, semi-arid region, the lake experiences a temperate continental monsoon climate characterized by distinct seasonal variations and significant diurnal temperature differences. The annual ice-cover period typically lasts from November to March of the following year. The mean annual precipitation in the region is 145 mm, which is relatively low and unevenly distributed, with over 60% of the total precipitation occurring during summer. The mean annual evaporation was 2092.3 mm. The average wind speed was 2.75 m/s, with a maximum recorded speed of 3.3 m/s; winds are generally strong and predominantly northwesterly. Annual solar radiation is considerable, with sunshine durations ranging from 2900 to 3200 h. Ulansuhai Lake has a total water storage capacity of 250–550 million m^3^. The average water depth was approximately 1.5 m, with water-level fluctuations causing this to vary between 0.5 m and 4 m. The multi-year average annual inflow into the lake is approximately 514 million m^3^, derived primarily from agricultural drainage in the Hetao Irrigation District and ecological water supplementation projects [18]. Lake water discharge occurs mainly through evaporation, followed by outflow to the Yellow River in the southern part of the lake, and subsurface seepage.

To capture the plankton community characteristics during the ecological water replenishment, field sampling was conducted twice: once in June 2023 and once in June 2024. Eight representative sampling sites (Q1–Q8) were strategically established across the major ecological and hydrological zones (Figure 1). The site selection followed the dominant north-to-south water flow gradient. Specifically, site Q1 (depth 2.0 m) is located at the primary inlet where the main drainage of the Hetao Irrigation District enters the lake. Q2 (depth 1.5 m) and Q3 (depth 1.6 m) serve as critical pathways for summer flash flood inputs. Sites Q4, Q5, and Q6 (mean depth 2.0 m) are open-water areas partitioned by extensive reed marshes, representing the central lake environment. Q7 (depth 1.8 m) is located in the southern open-water region, while Q8 (depth 1.7 m) is situated near the recession channel, marking the lake’s outlet. At each site, water samples were collected from the surface layer (0.5 m below the surface) for subsequent biological and physicochemical analyses.

### 2.2. Sample Collection Method

#### 2.2.1. Physicochemical Analysis

Water temperature (WT) and dissolved oxygen (DO) were measured in situ at each sampling site using a portable multiparameter water quality analyzer (YSI). Concurrently, water samples (1 L) were collected at a depth of 0.5 m below the surface using a water sampler. The collected samples were immediately transferred into pre-cleaned polyethylene bottles, stored in the dark at 4 °C in a portable cooler, and transported to the laboratory for physicochemical analyses within 24 h. In the laboratory, water quality parameters were determined in strict accordance with standard analytical protocols. Specifically, the permanganate index (COD_Mn_) and chemical oxygen demand (COD_Cr_) were determined using the standard permanganate method and the potassium dichromate oxidation method, respectively. The concentrations of total nitrogen (TN) and total phosphorus (TP) were measured using the alkaline potassium persulfate digestion UV spectrophotometric method and the ammonium molybdate spectrophotometric method, respectively. Furthermore, ammonia nitrogen (NH_3_-N) was quantified employing Nessler’s reagent spectrophotometry.

#### 2.2.2. Phytoplankton

Water samples were collected using an organic glass water sampler, and 1 L of water was collected and placed in a wide-mouth polyethylene bottle. A quantitative volume of Lugol’s solution was added at time of collection for fixation, after which the samples were returned to the laboratory and left undisturbed in the dark for 48 h. The supernatant was slowly removed using the siphon method, and the phytoplankton concentrate was transferred into sample bottles for preservation. A 0.1 mL of a well-mixed sample was placed in a Palmer-Maloney counting chamber. Species identification and cell counting were performed by observing 100 randomly selected fields of view under a light microscope at 10 × 40 magnification [19]. Phytoplankton abundance was expressed in units of cells·L^−1^, and biomass was estimated using the cell volume method (mg·L^−1^).

#### 2.2.3. Zooplankton

Zooplankton samples of cladocerans and copepods were collected in situ using 20-L water samples, which were filtered and concentrated through a 64 μm plankton net before being transferred to 100-mL stoppered polyethylene bottles. Quantitative Lugol’s solution and formalin were added on site for long-term fixation, after which the samples were transported to the laboratory for taxonomic identification and whole-sample microscopic counting [20]. Rotifers and protozoans were identified and counted using the phytoplankton samples [21]. For rotifer, the concentrated sample was thoroughly homogenized, and a 1 mL of subsample was transferred to a counting chamber. The entire chamber was examined under a microscope at 10 × 10 magnification, and each sample was counted three times. For protozoan, a 0.1 mL of subsample was transferred to a counting chamber and examined under a microscope at 10 × 40 magnification. Each sample was counted three times. Zooplankton biomass was estimated using taxon-specific length–weight regression equations and expressed as mg·L^−1^.

### 2.3. Calculation of Community Metrics

#### 2.3.1. Diversity Index

Shannon–Wiener index calculation:

$$H^{\prime }=-\sum_{i=1}^{s}\left(\frac{n_{i}}{N})ln(\frac{n_{i}}{N}\right)$$

Margalef Index Calculation:

D=(S−1)/lnN

Pielou index calculation:

$$J=\frac{H^{\prime }}{H_{max}}=\frac{H^{\prime }}{lnS}$$

where S is the number of species in the community, n_i_ is the number of individuals of the ith species, and N is the total number of individuals across all species.

#### 2.3.2. Dominant Species

Dominance was calculated based on the McNaughton dominance index (Y) using the following formula:

$$Y=\frac{n_{i}}{N}f_{i}$$

where ni represents the number of individuals of the i-th plankton species in the sample, N is the total number of all phytoplankton in the sample, fi is the frequency of occurrence of this species in each sample, and species with Y ≥ 0.02 are considered dominant species.

#### 2.3.3. Niche Breadth

The weighted modified Levins calculation formula by Colwell et al. was adopted to calculate niche breadth [22]:

$$B_{i}=\frac{1}{\sum_{j=1}^{r}{{(P}_{ij})}^{2}}$$

where *B*_*i*_ represents the niche breadth of the i-th species: P_*ij*_ is the proportion of species j in quadrat i, r is the number of quadrats, where *B*_*i*_ > 0.60 indicates a broad niche, 0.30 < *B*_*i*_ ≤ 0.60 indicates a medium niche, and *B*_*i*_ ≤ 0.30 indicates a narrow niche [23].

#### 2.3.4. Niche Overlap

The formula for the Pianka index calculation is [24]:

$$O_{iK}=\frac{\sum_{j=1}^{r}P_{ij}P_{kj}}{\sqrt{\sum_{j=1}^{r}{\left(P_{ij}\right)}^{2}}}$$

$${∆O}_{ik}=\sum_{k}O_{ik}-\sum_{i}O_{ik}$$

$$R=\frac{B_{i}}{∆O_{ik}}$$

where $O_{iK}$ represents the overlap value. P_ij_ and P_kj_ represent the proportions of individuals of species *i* and *k*, respectively, at site *j* relative to the total number of individuals of each species; and r is the total number of resource states. The value of $O_{iK}$ ranges from 0 to 1. When Δ$O_{iK}$ > 0, it indicates that the species is in a developmental phase; when Δ$O_{iK}$ < 0, the species is neutral, and R represents the ecological response rate.

#### 2.3.5. Overall Connectivity Test

The overall interspecific association was tested using Schluter’s variance ratio (VR) method [25]. Where VR > 1 indicated a net positive association, VR < 1 indicated a net negative association, and VR = 1 indicated no association. The statistical quantity W = VR × N was used to test the significance of the VR deviation from 1 using the following formula:

$$\delta_{T}^{2}=\sum_{i=1}^{S}P_{i}{\left(1-P_{i}\right)}^{2}$$

$$S_{T}^{2}=\frac{1}{n}\sum_{j=1}^{N}{(T}_{j}{-t)}^{2}$$

$$VR=\frac{\delta T^{2}}{ST^{2}}$$

where $\delta_{T}^{2}$ represents the variance of species relative abundance, S is the total number of dominant species, Pi = ni/N, where N is the number of quadrats and ni is the number of stations where species i occurs. $\delta_{T}^{2}$ denotes the variance of species number, Tj is the number of species in quadrat j, and t is the average number of species across all quadrats.

#### 2.3.6. Overall Interspecific Association of Species

Yate’s corrected chi-square statistic (χ^2^) was used to conduct connectivity and correlation analyses between species pairs [26].

(1) $$\chi 2=\frac{{\left(\left|ad-bc\right|-0.5n\right)}^{2}n}{\left(a+b)(a+c)(b+d)(c+d\right)}$$

where a, b, c, and d represent the co-occurrence and non-occurrence scenarios between the two: a is the number of quadrats where both species A and species B co-occur; b is the number of quadrats where only species A occurs; c is the number of quadrats where only species B occurs; d is the number of quadrats where neither species A nor species B occurs; and n is the total number of quadrats n = a + b + c + d.

The AC test refers to the degree of change and correlation between two variables, with values ranging from [−1, 1]. The formula is:

If ad ≥ bc:

$$AC=\frac{ad-bc}{\left(a+d)(b+d\right)}$$

If ad < bc, and d ≥ a:

$$AC=\frac{ad-bc}{\left(a+b)(a+c\right)}$$

If ad < bc, and d < a:

$$AC=\frac{ad-bc}{\left(c+d)(b+d\right)}$$

### 2.4. Statistical Analysis

All data in this study are presented as mean ± standard deviation (SD). Prior to statistical analysis, data normality and homogeneity of variance were assessed using the Shapiro–Wilk and Levene’s tests, respectively. Given that sampling was conducted at the same eight fixed sites, paired-samples *t*-tests (for normally distributed data) or the non-parametric Wilcoxon signed-rank test (for data not meeting normality assumptions) were employed to evaluate interannual differences in plankton density, biomass, and diversity indices. Principal Coordinates Analysis (PCoA) based on Bray–Curtis dissimilarity was performed to explore shifts in overall community composition. Niche differentiation was evaluated using Levins’ niche breadth index (B_i_) and Pianka’s niche overlap index (O_ik). For the interspecific association analysis of dominant species, a Yates-corrected chi-square (χ2) test was applied to determine the statistical significance of species co-occurrence, while the association coefficient (AC) was calculated to quantify the direction and intensity of these interactions. χ2 < 3.841 indicated a non-significant association (*p* > 0.05), 3.841 < χ2 < 6.635 indicated a significant association (0.01 < *p* < 0.05), and χ2 > 6.635 indicated a highly significant association (*p* < 0.01). The AC ranges from −1 to 1, where values closer to 1 represent stronger positive associations, values closer to −1 denote stronger negative associations, and AC = 0 signifies complete independence. To explore the interaction patterns and stability of the community, a plankton co-occurrence network was constructed based on Spearman’s rank correlation coefficient (retaining only edges with absolute correlation coefficients ∣r∣ > 0.7 and *p* < 0.05), and topological properties such as the number of nodes, the number of edges, average degree, and modularity were calculated. Network robustness was assessed by simulating random attacks (randomly removing 50% of the nodes) and targeted attacks (removing key nodes) and calculating the proportion of remaining species in the network. Additionally, positive and negative cohesion were calculated based on positive and negative abundance correlations, respectively, to quantify potential niche overlap or interspecific competition within the community. All statistical analyses and data visualizations were performed using R version 4.5.2.

## 3. Results

### 3.1. Water Replenishment

#### 3.1.1. Ecological Water Replenishment

**Figure 2:**
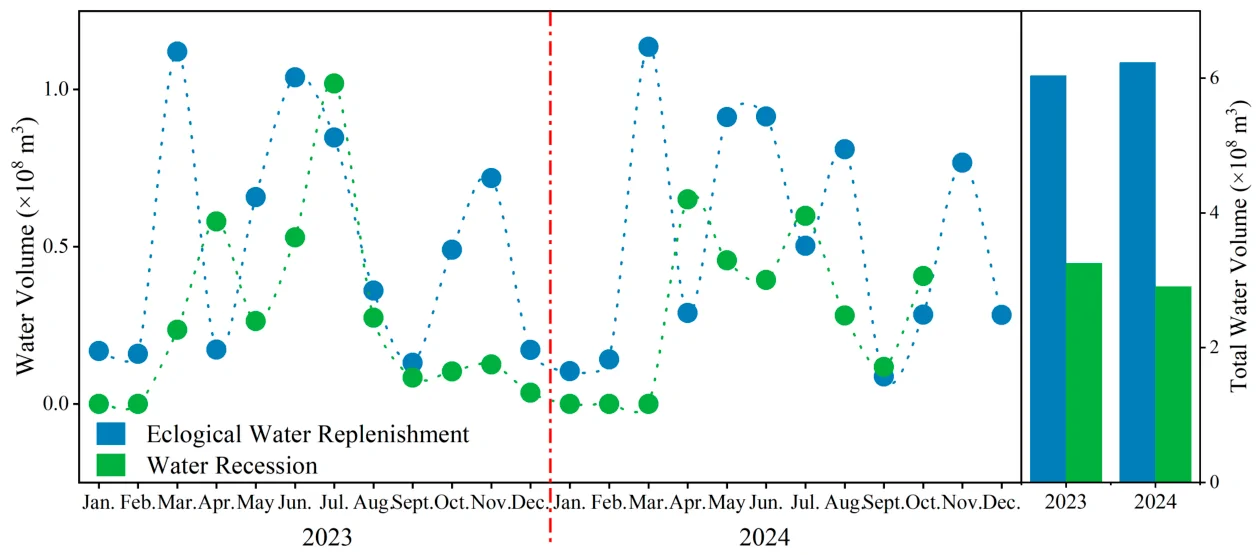
The amount of ecological water replenishment and recession.

The ecological water replenishment of Ulangsuhai Lake primarily relies on ice floodwater and temporary water supplementation upstream during the high-flow period (Figure 2). The ecological water replenishment volumes were 604 million m^3^ and 623 million m^3^, in 2023 and 2024, respectively, whereas the water discharge volumes for these years were 320 million m^3^ and 290 million m^3^, respectively, maintaining overall relative stability. In terms of intra-annual variations in water replenishment, ecological water supplementation was mainly concentrated in March, June, and November, corresponding to the irrigation period of the Hetao Irrigation District, flood season of the Yellow River, and the autumn irrigation period, respectively.

#### 3.1.2. Ecological Water Replenishment Water Quality Status

**Figure 3:**
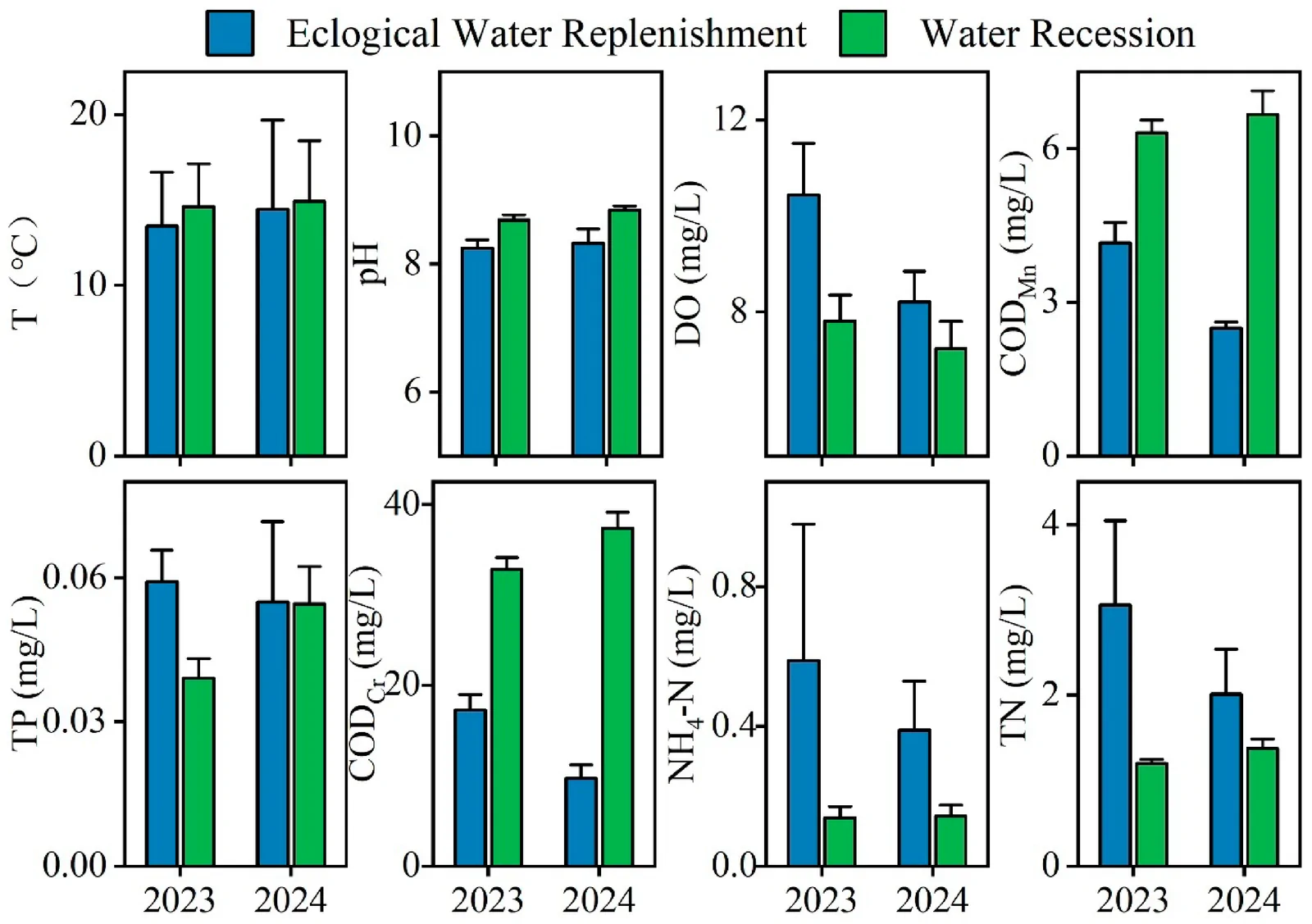
Key water quality indicators of ecological water replenishment and recession.

The quality of ecological water replenishment and water recession exhibited distinct water-quality characteristics, particularly in terms particularly with prominent indicators such as DO, COD_Cr_, COD_Mn_, and TN (Figure 3). The COD_Cr_, COD_Mn_, TN, TP, and NH_3_-N concentrations were 14.95 mg/L, 3.16 mg/L, 2.63 mg/L, 0.05 mg/L, and 0.23 mg/L, respectively. Notably, the TN concentration in the replenishment water (2.63 mg/L) significantly exceeded the Class V water limit (2 mg/L) (according to the surface water environmental quality standard (GB3838-2002) [27] (Figure S1). Based on the annual replenishment volume, the total nitrogen input into Lake Ulansuhai through ecological water replenishment is as high as 1776 tons per year, which may have a substantial impact on the eutrophication process occurring in the lake.

In contrast to ecological water replenishment, the water quality of recession displays distinct characteristics of pollution load transfer. The concentrations of COD_Cr_ and COD_Mn_ increased to 6.50 mg/L and 35.10 mg/L, respectively, and the TN and NH_3_-N decreased to 1.29 mg/L and 0.14 mg/L, respectively. The TP concentration remained relatively stable at 0.046 mg/L.

### 3.2. Plankton Community Composition

#### 3.2.1. Phytoplankton Species

**Figure 4:**
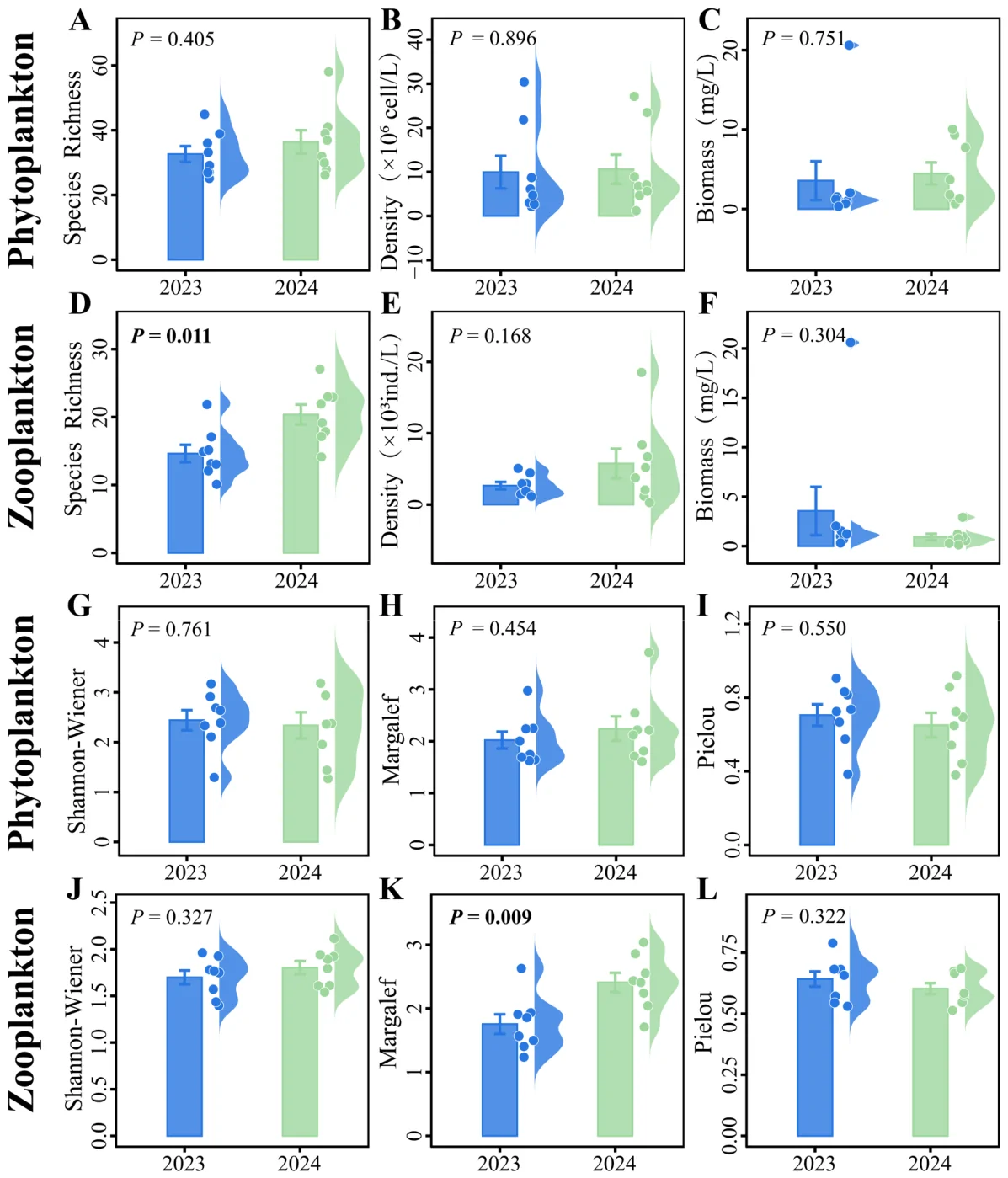
Variation in species number, density, biomass and diversity index of plankton. (**A**) Species richness of phytoplankton; (**B**) density of phytoplankton; (**C**) biomass of phytoplankton; (**D**) species richness of zooplankton; (**E**) density of zooplankton; (**F**) biomass of zooplankton; (**G**) shannon-Wiener index of phytoplankton; (**H**) margalef index of phytoplankton; (**I**) pielou index of phytoplankton; (**J**) shannon-Wiener index of zooplankton; (**K**) margalef index of zooplankton; (**L**) pielou index of zooplankton.

A total of 165 phytoplankton species belonging to 7 phyla were identified, with 122 and 88 species recorded in 2023 and 2024, respectively (Figure 4A). Cyanophyta, Chlorophyta, and Bacillariophyta are the dominant phyla, accounting for 85% and 88% of the total phytoplankton composition in 2023 and 2024, respectively. The remaining phyla (including Pyrrophyta, Chrysophyta, Euglenophyta, and Cryptophyta) collectively accounted for 15%, and 12%, respectively, in 2023 and 2024 (Figure S2). Differential analysis revealed no significant differences in phytoplankton species richness (2023: 25–45 species; 2024: 26–58 species), density (2023: 2.14–30.38 × 10^6^ cells/L; 2024: 1.19–27.12 × 10^6^ cells/L), or biomass (2023: 0.32–20.61 mg/L, average 3.56 mg/L; 2024: 0.63–10.05 mg/L, average 4.47 mg/L) in Lake Ulansuhai (*p* > 0.05) (Figure 4B,C).

#### 3.2.2. Zooplankton Species

A total of 64 zooplankton species, belonging to four groups (Copepoda, Cladocera, Roteria and Protozoa), were identified. Protozoa and Rotifera were the dominant components of the zooplankton community in terms of density (Figure S2B). Protozoa accounted for the largest proportion of total abundance at most sites, particularly in 2024. Rotifers maintained a consistently important role throughout the study period, although their relative proportions exhibited spatial fluctuations. In contrast, the density and biomass proportions of larger-bodied Cladocera and Copepoda remained relatively low at all sampling sites in both years, showing no significant interannual shifts (*p* > 0.05). The zooplankton community structure was characterized by a clear dominance of small-sized individuals (Protozoa and Rotifera), reflecting a community miniaturization under the current environmental background.

Differential analysis indicated a significant difference in zooplankton species richness between 2023 (10–22 species) and 2024 (14–23 species) (*p* < 0.05) (Figure 4D). However, no significant differences were observed in zooplankton density (2023: 2654.84 ind./L; 2024: 5758.1 ind./L) or biomass (2023: 0.32–20.61 mg/L, average 3.56 mg/L; 2024: 0.13–2.92 mg/L, average 0.94 mg/L) (*p* > 0.05) (Figure 4E,F). Rotifers and protozoans were the dominant groups in the zooplankton community structure, with rotifers maintaining a predominant role throughout the study period, albeit with slight fluctuations in their proportions. The proportions of cladocerans and copepods remained relatively low throughout the study period and showed no significant change.

#### 3.2.3. Variations in Plankton Community Diversity

During the study period, the Shannon–Wiener diversity index (H’) of phytoplankton communities in Lake Ulansuhai ranged from 1.27 to 3.18, while the Margalef diversity index (D) varied between 1.61 and 3.71. The Pielou evenness index ranged from 0.38 to 0.92 (Figure 4G–I). Differential analysis revealed that under high-nitrogen water replenishment conditions, the diversity indices of the phytoplankton communities did not exhibit significant changes (*p* > 0.05).

For the zooplankton communities in Lake Ulansuhai, the Shannon–Wiener diversity index (H’) fluctuated within a relatively narrow range of 1.40 to 2.12, with water quality characteristics classified as β-mesosaprobic (Figure 4J). The Pielou evenness index ranged from 0.51 to 0.79, indicating that although the Shannon and Pielou indices exhibited some degree of fluctuation, they did not reach statistical significance (*p* > 0.05) (Figure 4L). Further analysis revealed that the Margalef richness index (D) of zooplankton communities in Lake Ulansuhai averaged 1.75 and 2.41 in 2023 and 2024, respectively, showing an increasing trend, but this did not reach the level of significance (*p* > 0.05) (Figure 4K).

#### 3.2.4. Dynamics of Plankton Community Structure and Succession of Dominant Species

**Figure 5:**
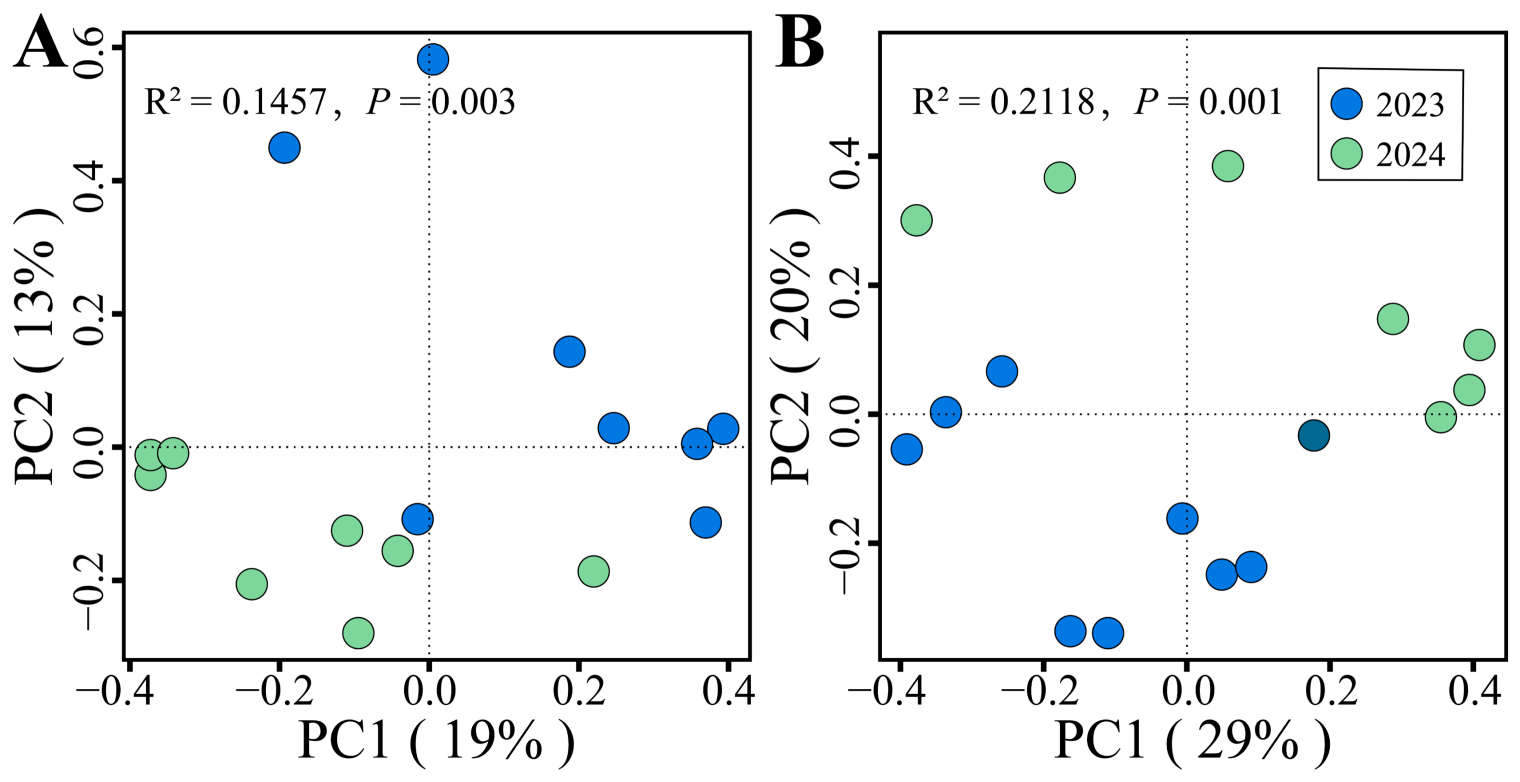
Differences in plankton community structure in the lake Ulansuhai. (**A**) Phytoplankton; (**B**) Zooplankton.

To further investigate the evolution of the plankton community structure in Lake Ulansuhai, we employed Principal Coordinate Analysis (PCoA) to explore changes in community composition. The results revealed significant differences in plankton community structure among the different groups in Lake Ulansuhai. For phytoplankton, the first principal component (PC1) and second principal component (PC2) explained 19% and 13% of the variation in community structure, respectively. Different phytoplankton species were distinctly distributed in different regions (R^2^ = 0.1457, *p* = 0.001), indicating that the phytoplankton community structure underwent significant changes under water replenishment conditions with high nitrogen content (Figure 5A).

For zooplankton, PC1 and PC2 explained 29% and 20% of community variation, respectively, demonstrating a relatively high overall explanatory power. Significant differences were observed among the sampling points from different years (R^2^ = 0.2688, *p* = 0.001), indicating notable succession in the zooplankton community (Figure 5B). From the perspective of dominant species shifts, the indicator species of phytoplankton transitioned primarily from Bacillariophyta to Cyanophyta and Chlorophyta, whereas those of zooplankton shifted from Rotifera to Cladocera and Copepoda (Table S1). A comprehensive analysis suggested that under water replenishment conditions with a high nitrogen content, the aquatic community structure in Lake Ulansuhai underwent significant succession. This phenomenon indicates that aquatic communities adapt to changes or disturbances in external environmental factors (e.g., water quality and nutrients) by adjusting their species composition.

### 3.3. Niche and Connectivity Analysis of Dominant Plankton Species

#### 3.3.1. Niche Breadth Analysis

Under high nitrogen supply conditions, notable shifts occurred in the dominant plankton community of Lake Ulansuhai (Table 1). In 2023, the dominant phytoplankton community in this water body comprised five species, which increased to six by 2024, among which *Merismopedia tenuissima* and *Pseudanabaena mucicola* from the phylum Cyanophyta were the dominant species. Concurrently, the dominant zooplankton community consisted of nine species in 2023, rising to 15 by 2024, including five species, including the protozoans *Strombidium sulcatum* and *Halteria grandinella*, as well as the rotifers *Brachionus angularis*, *Keratella quadrata*, and *nauplii*. Under high nitrogen supply conditions, the niche breadth of dominant phytoplankton species in Lake Ulansuhai ranged between 1.23 and 6.81, while that of dominant zooplankton species spanned 1.49 to 5.26, both exhibiting relatively broad niche distributions.

To further clarify the ecological habits and developmental status of dominant species under high-nitrogen replenishment, the ecological response rate (R) was calculated based on niche breadth (B_i_) and niche overlap (ΔO_ik_) (Table 1). All dominant phytoplankton species during the monitoring period were categorized as developmental types (R > 0). The Chlorophyta of *Scenedesmus quadricauda* (P1) exhibited the highest response rate (R = 15.38) and the broadest niche breadth (B_i_ = 6.82) in 2023, demonstrating a strong competitive advantage. By contrast, the dominance (Y) of the Cyanophyta of *Merismopedia minima* (P7) surged to 0.22 in 2024, with a corresponding response rate of R = 5.96, reflecting its rapid expansion under high-nitrogen conditions. Conversely, regarding zooplankton, *Keratella quadrata* (Z8) was the primary dominant species in 2023 (Y = 0.73, R = 9.50), yet its dominance significantly declined in 2024. The Rotifera of *Lecane papuana* (Z16) exhibited the highest ecological response rate (R = 10.86) among the zooplankton in 2024, indicating that the species possessed the greatest developmental potential during this period. Overall, the ecological response rates (R) for all dominant plankton species were positive (R > 0), indicating that the dominant populations were generally in a developmental phase, actively expanding their resource utilization space in response to the high-nitrogen environmental conditions.

#### 3.3.2. Niche Overlap Analysis

**Figure 6:**
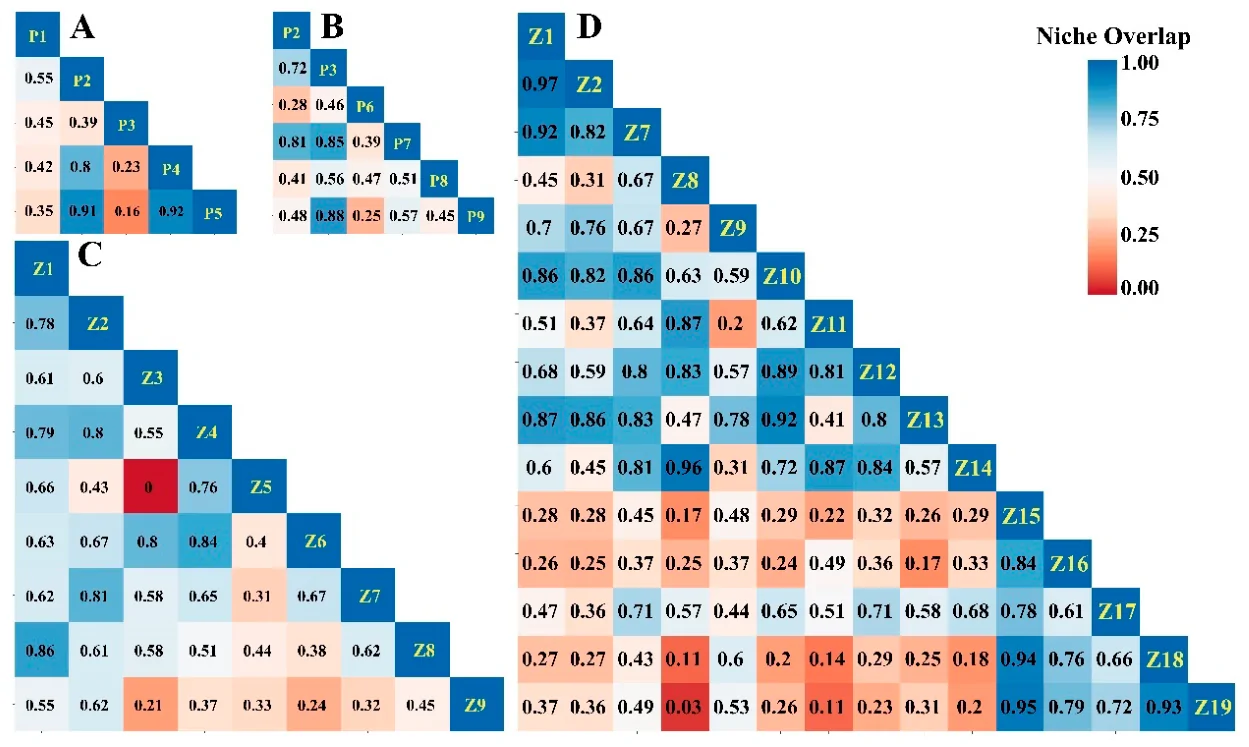
Niche overlap of dominant plankton species. The yellow font indicates the species codes, which correspond to the specific taxa listed in Table 1. (**A**) Phytoplankton in 2023; (**B**) phytoplankton in 2024; (**C**) zooplankton in 2023; (**D**) zooplankton in 2024.

Niche overlap value (O_ik_) is a crucial indicator of the degree of resource competition among plankton species. In this study, the phytoplankton niche overlap values (O_ik_) in Lake Ulansuhai for 2023 were calculated and revealed a range of [0.159–0.921], with an average of 0.518 (Figure 6A). Compared to the control group’s average niche overlap value of 0.65, seven pairs of species exhibited overlap values below 0.6, accounting for 33.3% of the total pairs, whereas three pairs exceeded 0.8, primarily P2 and P4 (0.8), P2 and P5 (0.91), and P4 and P5 (0.96). In 2024, the phytoplankton niche overlap value (O_ik_) for Lake Ulansuhai ranged from [0.253, 0.882], with an average of 0.540 (Figure 6B). Among these, 11 pairs of species had overlap values below 0.6, with the minimum observed between P6 and P9 (0.253), and the maximum between P3 and P9.

For the zooplankton niche overlap values (O_ik_) in Lake Ulansuhai in 2023, the range is [0, 0.857], with an average of 0.556. A total of 18 pairs of species exhibited overlapping values below 0.6, with the minimum value observed between Z3 and Z5 (0) (Figure 6C). In 2024, the zooplankton niche overlap values (O_ik_) in Lake Ulansuhai will range from [0.035, 0.966], with an average of 0.535 (Figure 6D). Sixty pairs of species had overlap values below 0.6, accounting for 57%, with a minimum between Z8 and Z19 and a maximum between P3 and P9. The analysis indicated that under high-nitrogen water replenishment conditions, the niche overlap values of plankton in Lake Ulansuhai remained relatively stable.

To further investigate the ecological habits and developmental trends of dominant plankton species in Lake Ulansuhai under high-nitrogen water replenishment, this study calculated the ΔO_ik_ and ecological response rate of dominant species in combination with niche width (Table 2). ΔO_ik_ values of dominant phytoplankton species in Lake Ulansuhai in both 2023 and 2024 were consistently below 0, revealing that phytoplankton are in a declining phase under high-nitrogen water replenishment conditions. Additionally, the ΔO_ik_ values of zooplankton were lower than those of phytoplankton, indicating a more pronounced declining trend in plankton under high-nitrogen water replenishment conditions.

#### 3.3.3. Dominant Species Overall Correlation Analysis

Under high nitrogen supply conditions, the species richness index (VR value) of the plankton community in Ulansuhai was >1, revealing a prevalent positive correlation among the dominant species. Through the test of the statistical value W, we found that the overall association of phytoplankton dominant species during high nitrogen supply was significant (W > $\chi_{0.95}^{2}$(df)), indicating a relatively tight connection among dominant species, and the community structure of phytoplankton exhibited high stability. The impact of high nitrogen supply on the stability of the phytoplankton community was relatively minor. Conversely, under high nitrogen supply conditions, the overall positive association of zooplankton dominant species in Ulansuhai was not significant ($\chi_{0.95}^{2}$(df) < W < $\chi_{0.05}^{2}$(df)), suggesting the loose interspecific connection among zooplankton dominant species and the instability of the community structure. In addition, external disturbances had a greater impact on the zooplankton community stability (Table 2).

#### 3.3.4. Interspecific Associations

**Figure 7:**
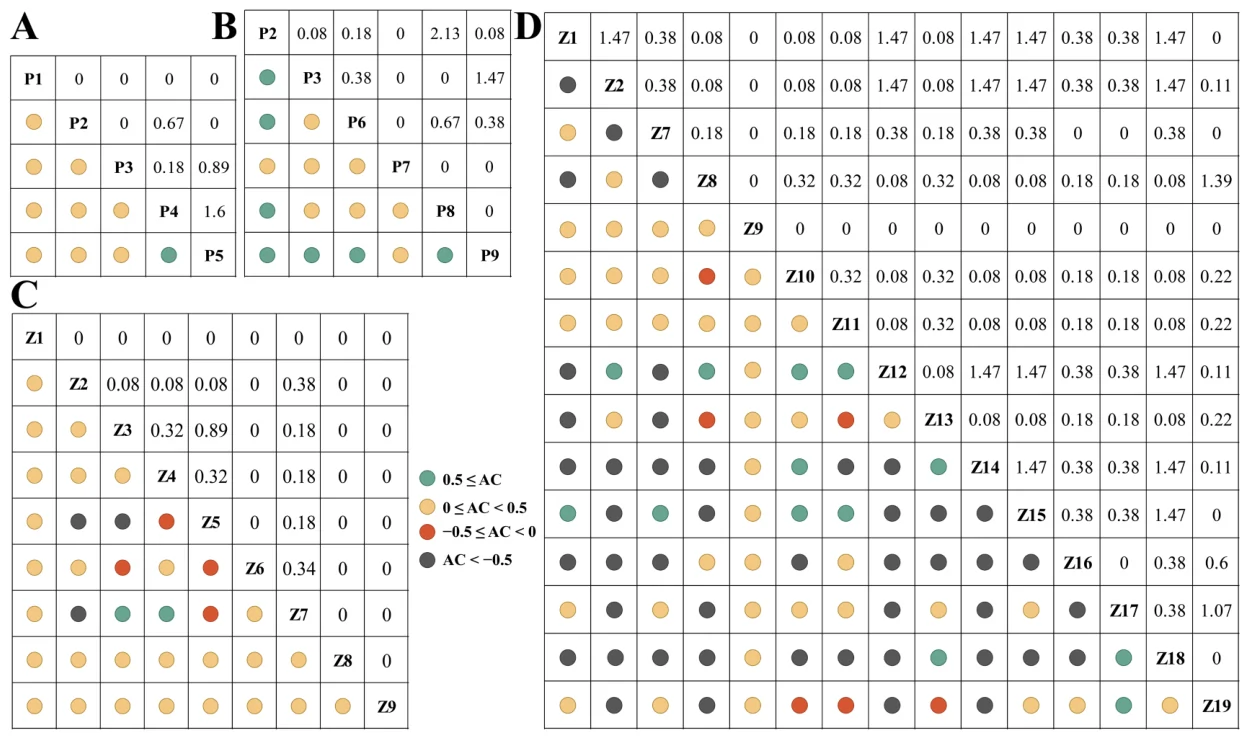
Interspecific association of plankton dominance. Numbers represent the results of the χ2 test, and graphs represent the semi-matrix diagram of the interspecific association (AC). (**A**) Phytoplankton in 2023; (**B**) phytoplankton in 2024; (**C**) zooplankton in 2023; (**D**) zooplankton in 2024.

Based on the χ2 test of dominant species associations, under high nitrogen water supplementation conditions, there were 25 pairs of dominant phytoplankton species in Lake Ulansuhai, while the interspecific associations among species were not significant (χ2 < 3.841). However, the association coefficient revealed that in 2023, five pairs of phytoplankton exhibited positive associations (AC > 0), with P1 being completely antagonistic to other species (Figure 7A). In 2024, among the phytoplankton, except for P7, which was completely independent of the other species, most species pairs showed positive associations, accounting for 60% of the total (Figure 7B). In 2023, seven pairs of zooplankton displayed positive associations (AC > 0), whereas 21 pairs were completely antagonistic (Figure 7C). In 2024, 38 pairs of zooplankton exhibited positive associations, representing a relatively high proportion, with Z9 remaining completely antagonistic to other species (Figure 7D). Therefore, under high-nitrogen water supplementation, phytoplankton maintained positive associations, whereas zooplankton exhibited a higher proportion of negative associations, indicating community fluctuations.

#### 3.3.5. Co-Occurrence Network Characteristics and Ecological Stability

**Figure 8:**
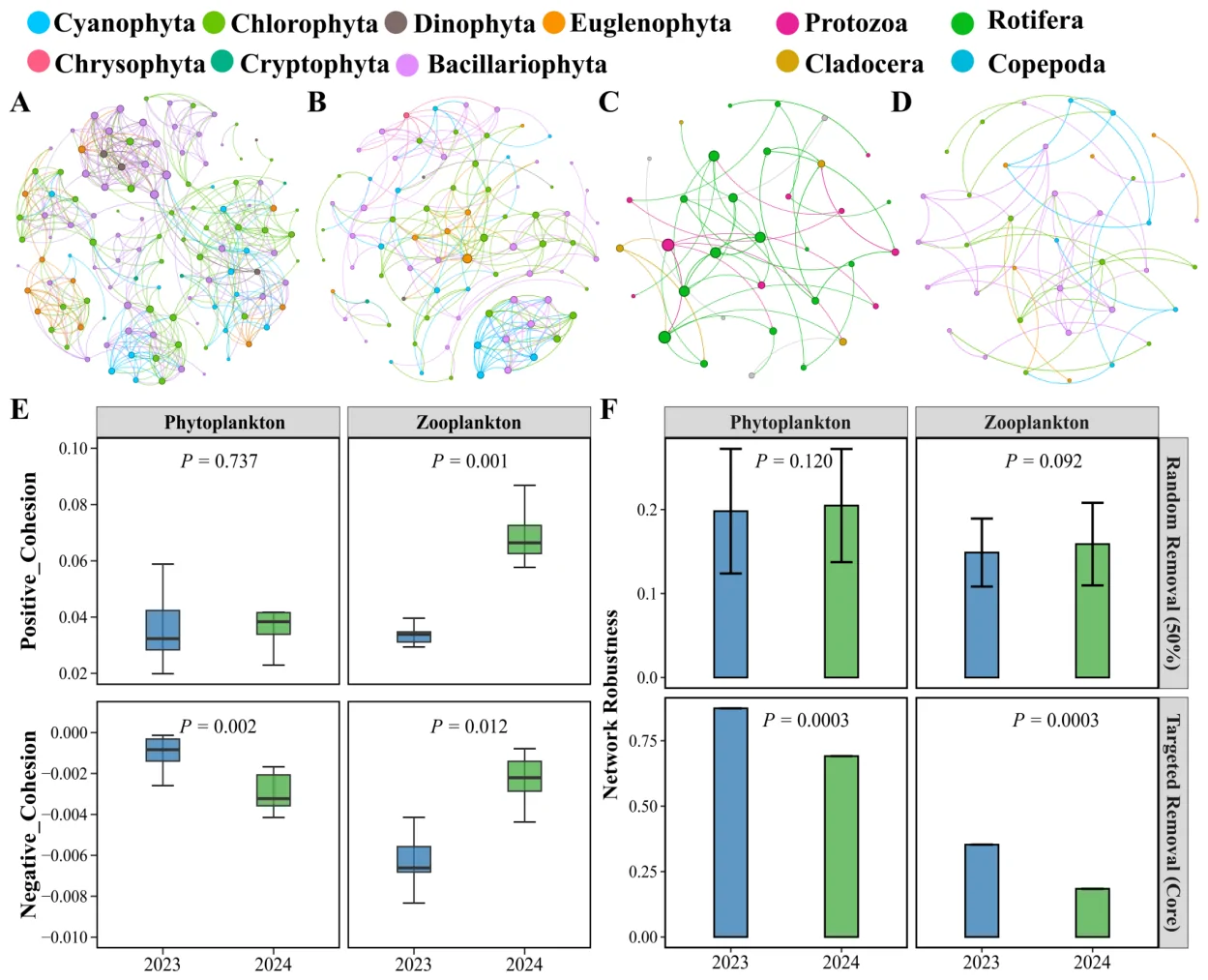
Co-occurrence networks and structural characteristics of plankton communities in Lake Ulansuhai. (**A**) Phytoplankton in 2023; (**B**) phytoplankton in 2024; (**C**) zooplankton in 2023; (**D**) zooplankton in 2024; (**E**) interannual comparison of positive and negative cohesion; (**F**) network robustness under random removal (50%) and targeted removal (core nodes).

Co-occurrence network analysis revealed that both phytoplankton and zooplankton formed complex association networks characterized by high modularity (with modularity values exceeding 0.72), though their network topologies differed in certain respects (Table S2, Figure 8). Overall, the phytoplankton network was larger and more complex than the zooplankton network. More specifically, the phytoplankton co-occurrence network was largest and most tightly connected in 2023 (119 nodes, 504 edges, and an average degree of 8.47); whereas in 2024, the topology was significantly simplified (the number of nodes dropped to 81, the number of edges plummeted to 223, and the average degree fell to 5.51), indicating a sharp decline in interactions among species. In contrast, the zooplankton network remained relatively small over the two years but showed a slight trend toward expansion: the number of nodes (increasing from 34 to 38), the number of edges (increasing from 46 to 59), and the average degree (rising slightly from 2.71 to 3.11) all increased, indicating a slight strengthening of interspecies connections.

Network cohesion analysis further revealed the dynamics of interspecific interactions underlying these topological changes (Figure 8E). For positive cohesion, which reflects potential cooperation or niche sharing, there was no significant change in phytoplankton over the two years (*p* = 0.737), whereas zooplankton showed a significant increase in 2024 (*p* < 0.001). Concerning negative cohesion, which reflects competitive interactions, phytoplankton showed a significant decrease in 2024 (i.e., an increase in absolute value, *p* = 0.002), indicating that interspecific competition intensified as the network simplified; conversely, zooplankton exhibited a significant increase in negative cohesion in 2024 (i.e., a decrease in absolute value, *p* = 0.012), reflecting a relative easing of competitive interactions. Robustness analysis reveals that, under conditions where 50% of the nodes were randomly removed, there were no significant interannual differences in the maximum connectivity ratio of either network over the two-year period (Figure 8F). However, under directed attacks involving the removal of key nodes, the robustness of both the phytoplankton and zooplankton networks declined significantly in 2024, and the overall robustness of phytoplankton remained consistently higher than that of zooplankton. The sharp decline in robustness under targeted attacks indicates that key species play a decisive role in maintaining network connectivity.

## 4. Discussion

### 4.1. Evolution Characteristics of the Plankton Community Structure of Broussonetia papyrifera Under High Nitrogen Supply Conditions

Under high nitrogen supply conditions, the species richness, density, and biomass of the plankton community showed no significant interannual variations (*p* > 0.05), indicating that the plankton in Lake Ulansuhai maintained a relatively stable state. A key factor contributing to this phenomenon lies in the fact that Lake Ulansuhai is a typical macrophyte-dominated lake with unique ecological regulation mechanisms. Aquatic vegetation with high overjet rates (e.g., *Phragmites australis*, *Potamogeton pectinutus* and *Myriophyllum verticillatum*) can absorb large amounts of nutrients, such as nitrogen and phosphorus, thereby reducing the nutrient concentrations in the water [28,29,30]. In contrast, the annual harvesting of aquatic plants removes nutrients, forming an effective nutrient export pathway [31] (Figure 3). This approach partially mitigates the external effects of a high nitrogen supply and maintains a relatively stable nutrient balance. Other studies have also confirmed that biological regulation measures, such as harvesting of aquatic plants, can enable large-scale safe reuse of reclaimed water while minimizing ecological risks to receiving water bodies [32]. Monitoring data indicated that under ecological supply conditions, nitrogen levels in Lake Ulansuhai did not increase significantly, effectively mitigating the impact of high nitrogen supply on aquatic organisms [33].

Furthermore, this study found that under high nitrogen supply conditions, the diversity indices of the biological community in Lake Ulansuhai remained relatively stable, without significant fluctuations. Community diversity indices are key indicators of community and ecosystem health and stability [34,35]. Previous studies have shown that water replenishment often leads to a decline in aquatic biodiversity indices and may even trigger ecosystem collapse. This further demonstrates that the biological community of Lake Ulansuhai has maintained good biodiversity, reflecting the overall favorable health of the water environment under the current supply conditions [36]. However, we emphasize that the absence of significant fluctuations in the overall community diversity index (*p* > 0.05) does not indicate no changes in community structure. As shown by the PCoA results, structural shifts have occurred in the internal composition of the plankton community, and changes have been observed in the relative abundances of dominant species. This demonstrates that, although biological indicators exhibit relative consistency under the current water replenishment conditions, the community is responding to environmental changes through reassembly.

### 4.2. Analysis of Plankton Niche Characteristics Under High Nitrogen Replenishment Conditions

In ecological research, niche breadth is a key indicator for measuring species adaptation to resource utilization and environmental changes. It reveals the position of a species within the ecosystem and its distribution patterns [37,38,39]. An increase in niche breadth typically signifies that a species has a broader range of resource utilization, a more uniform distribution pattern, and greater environmental adaptability [40,41]. This study conducted an in-depth analysis of the plankton niche characteristics in Lake Ulansuhai under high nitrogen replenishment conditions. These results indicate that the dominant populations exhibit larger niche breadths, suggesting that these species possess significant advantages in terms of resource utilization and environmental adaptation [42,43]. This phenomenon is primarily attributed to the alteration of the water nutrient structure owing to high nitrogen input, which subsequently drives adaptive adjustments in the plankton community. Specifically, under high nitrogen replenishment conditions, certain phytoplankton and zooplankton species expand their niches (e.g., by utilizing different forms of nitrogen sources or adjusting feeding strategies) to adapt to the new nutrient environment, thereby occupying broader ecological spaces within the community. Furthermore, the increase in niche breadth reflects the high plasticity of the plankton community in Lake Ulansuhai under high nitrogen replenishment conditions. These organisms maintain ecosystem stability by adjusting their species composition and functionality. PCoA results also demonstrated changes in the plankton community structure. Therefore, it could be inferred that long-term high nitrogen inputs may drive the plankton community toward more generalized resource utilization strategies, thereby exerting a profound impact on the structure and function of the entire lake ecosystem.

Interspecific associations are important indicators of the stage and stability of community succession. Specifically, the frequent occurrence of negative associations in a community signifies its early succession stages. As succession progressed, positive associations among species gradually increased, and an increase in the proportion of positive associations indicated the community’s progression toward a stable state, where dynamic equilibrium was achieved among species, manifesting significant positive correlations [44,45]. This study found that under high nitrogen level water replenishment conditions, the positive correlations among phytoplankton were significantly enhanced, whereas the correlations among zooplankton did not reach statistical significance, reflecting the tightness of phytoplankton relationships and the looseness of zooplankton relationships. The underlying cause of this phenomenon lies in lake ecosystems, where zooplankton, as secondary producers, have community structures that are largely dependent on the species composition and biomass of phytoplankton [46]. Under high nitrogen-level water replenishment conditions, phytoplankton communities may exhibit strengthened positive interspecific associations, owing to the remission of resource competition (e.g., sufficient nitrogen sources). Conversely, zooplankton associations may appear weaker because of feeding selectivity (e.g., preference for specific algae) or interspecific competition (e.g., resource competition between cladocerans and copepods). Additionally, the life history strategies of zooplankton (e.g., migratory behavior and formation of resting eggs) may increase the uncertainty of their community dynamics, further diminishing the significance of interspecific associations. Therefore, the findings of this study reveal that under high nitrogen input conditions, phytoplankton communities tend toward stability, whereas those of zooplankton remain in a phase of dynamic adjustment. This finding may have significant implications for the ecosystem functioning of Lake Ulansuhai, and future research should incorporate long-term observational data to further validate this conclusion.

### 4.3. Features of Interaction in the Plankton Network Under High-Nitrogen Supplementation

The co-occurrence network in aquatic ecosystems not only reflects the patterns of co-occurrence among taxa but also provides insights into the ecological interaction within the community [47,48]. The network analysis in this study shows that the size and complexity of the phytoplankton network are, on the whole, far greater than those of the zooplankton network. This phenomenon mainly stems from their different trophic levels within the food web. Zooplankton, as secondary producers, have a community structure that relies extensively on the species composition and biomass of phytoplankton [49,50,51]. Moreover, the size-mediated nature of zooplankton grazing—characterized by differences in feeding rates, prey selectivity, and dispersal across size classes—leads to complex niche overlaps and competition that shape the resulting network structure [52]. Coupled with flexible life-history adjustments (e.g., dormancy vs. development) in response to environmental fluctuations, these factors induce pronounced spatiotemporal variability in population dynamics [53]. Such inherent instability likely weakens interspecific connectivity, leading to the formation of relatively small, loose, and fragmented network structures.

Further analysis combining network cohesion and robustness revealed that phytoplankton and zooplankton communities underwent distinct restructuring processes. Generally, positive correlations within co-occurrence networks signify niche overlap and shared environmental preferences, whereas negative correlations characterize competitive or antagonistic interactions [54]. Regarding the phytoplankton community under high-nitrogen replenishment in 2024, positive cohesion remained stable (*p* = 0.737), whereas negative cohesion strengthened significantly (*p* = 0.002). This indicates that under nitrogen-saturated conditions, phytoplankton potentially intensified competition for other limiting resources, such as light or phosphorus [55]. Conversely, the zooplankton network exhibited a significant increase in positive cohesion (*p* < 0.001) and a concomitant weakening of negative cohesion (*p* = 0.012) in 2024. These shifts likely reflect a “bottom-up” regulatory effect, where the structural configuration of the zooplankton network is predominantly driven by changes in the phytoplankton community [52,56]. Specifically, zooplankton may have adapted to altered phytoplankton compositions by increasing their shared preference for dominant algal resources, thus reconfiguring internal community relationships. Furthermore, robustness analysis demonstrated that the overall stability of phytoplankton consistently exceeded that of zooplankton. This disparity is primarily attributed to the broader niche breadth and superior environmental adaptability of phytoplankton, which allow them to better buffer against fluctuations in environmental pressure [57]. Taken together, these findings suggest that plankton communities are undergoing profound dynamic adjustments and structural reorganization under high-nitrogen water replenishment, a process that redefines interspecific interaction patterns and influences overall network stability.

### 4.4. Study Limitations and Ecological Implications

Although this study preliminarily explored the changes in plankton communities under high-nitrogen water replenishment, several limitations must be explicitly acknowledged. First, the study is limited to two sampling events (June 2023 and June 2024) and lacks long-term observational data. Consequently, it cannot establish a causal relationship between high-nitrogen replenishment and community shifts, nor can it systematically evaluate the long-term ecological stability, resistance, or resilience of the ecosystem. Second, stability encompasses multiple dimensions, including temporal variability, functional redundancy, and ecosystem functioning, none of which were assessed in this study. To comprehensively elucidate the mechanisms driving plankton dynamics and robustly evaluate the integrated ecological risks versus water-resource benefits of ecological replenishment, future research must incorporate large-scale, multi-year, and multi-season monitoring, thereby effectively supporting lake water resource management and ecological conservation.

## 5. Conclusions

Under the background of high-nitrogen ecological water replenishment, the plankton communities in Lake Ulansuhai underwent distinct structural shifts between the 2023 and 2024 sampling periods, although overall community metrics (species richness, density, biomass, and diversity indices) showed no significant differences. Plankton communities exhibit adaptive niche widening and asynchronous dynamics. Niche and interspecific association analyses indicated that the plankton communities exhibited broad niche characteristics and different interspecific associations, reflecting the differentiated responses of species to environmental conditions. Co-occurrence network analysis further confirmed that, although the community maintained a highly modular network structure, communities in 2024 experienced a reduction in network size, intensified interspecific competition (increased negative cohesion), and decreased robustness in response to targeted removal. This study illuminated structural changes in plankton community under high-nitrogen ecological water replenishment from the perspectives of population distribution and community interactions. However, this study is limited to two sampling periods and does not compare alternative replenishment strategies. To comprehensively understand the response mechanisms of lake ecosystems to such replenishment strategies, large-scale, long-term observational and experimental studies are essential. Future research should integrate comprehensive assessments of ecological risks versus water-resource benefits to robustly support regional water resource management and ecological conservation efforts.

## Figures and Tables

**Table 1:** Dominance, niche width and ecological response rate of plankton dominant species.

Taxa		Species	Code	Y		Niche Width		Niche Overlap		R	
				2023	2024	2023	2024	2023	2024	2023	2024
Phytoplankton	Chlorophyta	*Scenedesmus quadricauda*	P1	0.02		6.82		0.44		−1.64	
		*Scenedesmus armatus*	P9		0.03		3.51		0.53		−2.84
	Cyanophyta	*Merismopedia tenuissima*	P2	0.03	0.04	2.75	1.49	0.66	0.54	0.12	−2.70
		*Pseudanabaena mucicola*	P3	0.02	0.03	3.04	5.26	0.31	0.69	−2.73	−1.16
		*Gomphosphaeria* sp.	P4	0.08		1.78		0.59		−0.44	
		*Coelosphaerium* sp.	P5	0.05		1.23		0.59		−0.50	
		*Aphanizomenon* sp.	P6		0.07		3.03		0.37		−4.38
		*Merismopedia. minima*	P7		0.22		3.73		0.63		−1.84
		*Microcystis densa*	P8		0.10		1.94		0.48		−3.30
Zooplankton	Protozoa	*Strobilidium gyrans*	Z1	0.43	0.24	6.31	3.43	0.69	0.59	−9.00	−39.74
		*Strombidium viride*	Z2	0.19	0.30	5.10	3.29	0.66	0.53	−9.40	−41.23
		*Askenasia colvox*	Z3	0.03		2.23		0.56		−12.19	
		*Didinium balaianilnanum*	Z4	0.03		2.63		0.66		−9.50	
		*Didinium nasutum*	Z5	0.03		1.54		0.47		−13.39	
		*Strobilidium velox*	Z10		0.07		3.90		0.61		−38.99
		*Sphaerophrya solifomis*	Z11		0.03		3.70		0.48		−42.64
		*Epistylis plicatilis*	Z12		0.06		5.16		0.62		−38.71
		*Vorticella* sp.	Z13		0.02		3.77		0.58		−40.01
	Copepoda	Nauplius	Z9	0.06	0.16	3.60	4.77	0.39	0.52	−13.86	−39.24
		Copepodid	Z18		0.11		3.48		0.43		−44.07
		*Eucyclops serrulatus*	Z19		0.02		2.99		0.45		−43.57
	Rotifera	*Brachionus calyciflorus*	Z6	0.03		1.84		0.58		−10.77	
		*Brachionus angularis*	Z7	0.04	0.02	3.83	4.26	0.57	0.68	−10.87	−37.21
		*Keratella quadrata*	Z8	0.73	0.04	5.27	2.83	0.55	0.47	−11.15	−42.97
		*Keratella ualga*	Z14		0.24		3.19		0.56		−40.55
		*Brachionus quadridentatus*	Z15		0.03		3.61		0.47		−43.06
		*Lecane papuana*	Z16		0.02		4.70		0.43		−44.01
	Cladocera	*Pleuroxus hamulatus*	Z17		0.02		3.95		0.60		−39.24

**Table 2:** Overall association analysis of dominant plankton species.

Plankton	Group	N	S	VR	W	[$\chi_{0.95}^{2}$(df) ≤ χ^2^ ≤ $\chi_{0.05}^{2}$(df)]	Community Relevance
Phytoplankton	2023	8	5	2.14	17.1	[2.73,15.51]	Positive association
	2024	8	6	2.79	22.32	[2.73,15.51]	Positive association
Zooplankton	2023	8	9	1.15	9.2	[2.73,15.51]	Not Significantly Positively Correlated
	2024	8	15	1.73	13.82	[2.73,15.51]	Not Significantly Positively Correlated

## Data Availability

The original contributions presented in the study are included in the article. The data presented in this study are available on request from the corresponding author.

## Supplementary Materials

The following supporting information can be downloaded at https://www.mdpi.com/article/10.3390/biology15201814/s1, Figure S1: Monthly variations of total nitrogen (TN) concentration during ecological water replenishment and water recession from 2023 to 2024; Figure S2: Variation in the relative abundance of plankton community composition in different sampling sites (A. Phytoplankton, B. Zooplankton); Table S1: The relative abundances of plankton dominant species; Table S2: Topological properties of plankton co-occurrence networks in Lake Ulansuhai.
