# Supplementary material for: Response of Plankton Community Under High-Nitrogen Ecological Replenishment: Niche Differentiation and Species Interactions
Source: Biology (Basel). 2026 Oct 11;15(20):1814. doi: 10.3390/biology15201814 (PMC13701269; doi:10.3390/biology15201814)
Supplement: Supplementary file 1 [file biology-15-01814-s001.zip › biology-4479008-supplementary.pdf]

Response of Plankton Community Under High-Nitrogen Ecological Replenishment: Niche Differentiation and Species Interactions

Caili Du [1,†], Tao Wu [1,2,†], Zhenjun Tian [1], Jingxuan Wang [1], Xianzhe Zhang [1], Zhaozhi Liu [1], Jianglong Cui [1], Shengwang Gao [1] and Lieyu Zhang [1,*]

[1] State Key Laboratory of Environmental Criteria and Risk Assessment, Chinese Research Academy of Environmental Sciences, Beijing, 100012, China
[2] Department of Environmental Science and Engineering, Fudan Unersity, Shanghai 200433, P.R. China
*Corresponding author: Lieyu Zhang
e-mail: zhanglieyu@163.com;
† These authors contributed equally to this work.

Competing: Interests.

The authors declare that they have no conflict of interest.

Supplementary material

Table S1. The relative abundances of plankton dominant species.

Taxa		Species	2023	2024
Phytoplankton	Bacillariophyta	*Cocconeis placentula*	0.06%	0.26%**
		Dictyosphaerium sp.	-	1.52%**
		Achnanthes inflata	-	0.26%**
		Cyclotella catenata	0.02%	1.56%**
		Amphora sp.	0.02%	0.14%**
		Cocconeis placentula var.lineate	0.06%	0.16%**
	Pyrrophyta	*Gymnodinium mitratum*	0.34%**	-
	Cyanophyta	*Synechocystis willei*	0.20%**	-
		Raphidiopsis sp.	-	1.15%**
		Merismopedia minima	6.61%	21.90%**#
		Aphanizomenon sp.	-	9.37%**#
		Pseudanabaena mucicola	3.86%#	3.72%**#
		Spirulina sp.	-	0.16%**
		Microcystis densa	-	20.38%**#
		Merismopedia tenuissima	4.04%#	9.51%#
		Gomphosphaeria sp.	32.69%#	-
		Coelosphaerium sp.	12.06%#	0.76%
	Chlorophyta	*Chlamydomonas ovalis*	0.49%**	-
		Kirchneriella contorta	0.18%**	-
		Scenedesmus quadricauda	2.71%**#	0.85%
		Ankistrodesmus falcatus	0.86%**	0.36%

		Tetraedron minimum	0.55%**	0.04%
		Scenedesmus armatus	-	3.52%**#
		Colenkinia radiata	-	0.27%**
		Nephrocytium sp.	-	0.19%**
		Kirchneriella sp.	0.47%	0.47%**
		Scenedesmus dimorphus	0.57%	0.95%**
		Tetraedrom trilobulatum	0.04%	0.13%**
		Tetraedrom pusillum	0.45%	0.67%**
	Cryptophyta	*Chroomonas caudata*	0.34%**	-
		Cryptomonas ovata	0.36%**	0.14%
Zooplankton	Rotifera	*Keratella quadrata*	73.09%**#	6.71%#
		Lecane papuana	-	3.00%**#
		Asplanchna sp.	0.05%	1.96%**
		Monostyla cornuta	-	1.96%**
		Keratella ualga	0.25%	27.37%#
		Brachionus calyciflorus	6.50%#	5.07%
		Brachionus angularis	4.94%#	2.94%#
		Brachionus quadridentatus	1.10%	3.54%#
	Copepoda	Nauplius	5.6%*#	16.25%#
		Eucyclops serrulatus	-	3.22%**#
		Copepodid	1.75%	12.92%#
	Protozoa	*Strobilidium gyrans*	42.63%**#	27.59%#
		Epistylis plicatilis	-	6.40%**#
		Sphaerophrya solifomis	-	4.05%**#
		Vorticella sp.	-	3.38%**#
		Holophrya atra	-	3.68%*
		Strobilidium velox	1.90%	11.26%**#
		Strombidium viride	21.61%#	33.92%**#
		Askenasia colvox	7.01%#	-
		Didinium balaianilnanum	8.61%#	2.35%
		Didinium nasutum	7.74%#	0.22%
	Cladocera	*Pleuroxus hamulatus*	-	2.94%**#
		Pleuroxus laevis	-	0.71%**

Note: #, dominant species; * & **, indicator species, *P < 0.05, ** P < 0.01; −, not detected in the group.

Table S2. Topological properties of plankton co-occurrence networks in Lake Ulansuhai.

Plankton	Group	Nodes	Edges	Degree	Avg_Path_Length	Density	Modularity
Phytoplankton	2023	119	504	8.47	5.13	0.07	0.75
	2024	81	223	5.51	2.90	0.07	0.72
Zooplankton	2023	34	46	2.71	1.94	0.08	0.76
	2024	38	59	3.11	2.35	0.08	0.73

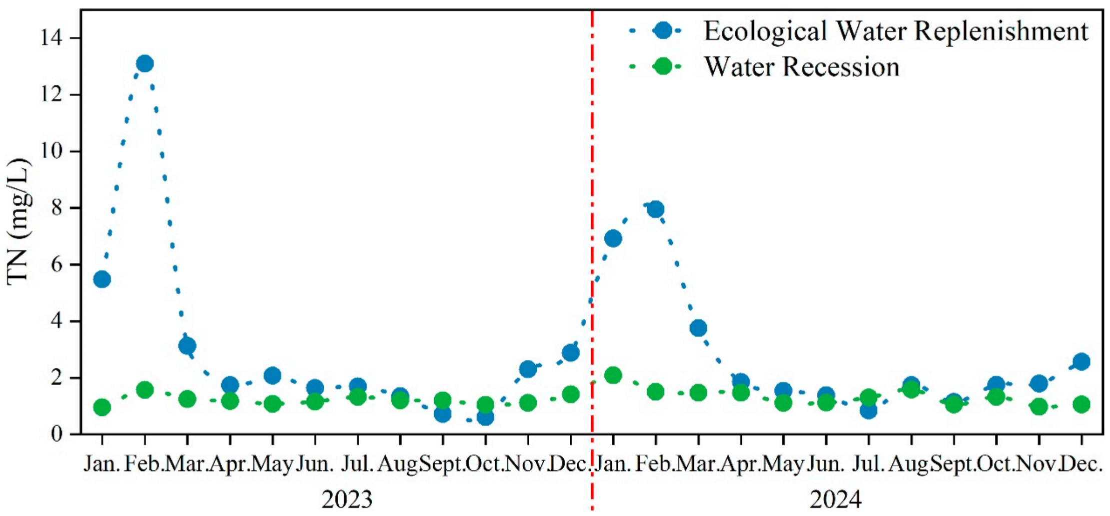

Figure S1. Monthly variations of total nitrogen (TN) concentration during ecological water replenishment and water recession from 2023 to 2024.

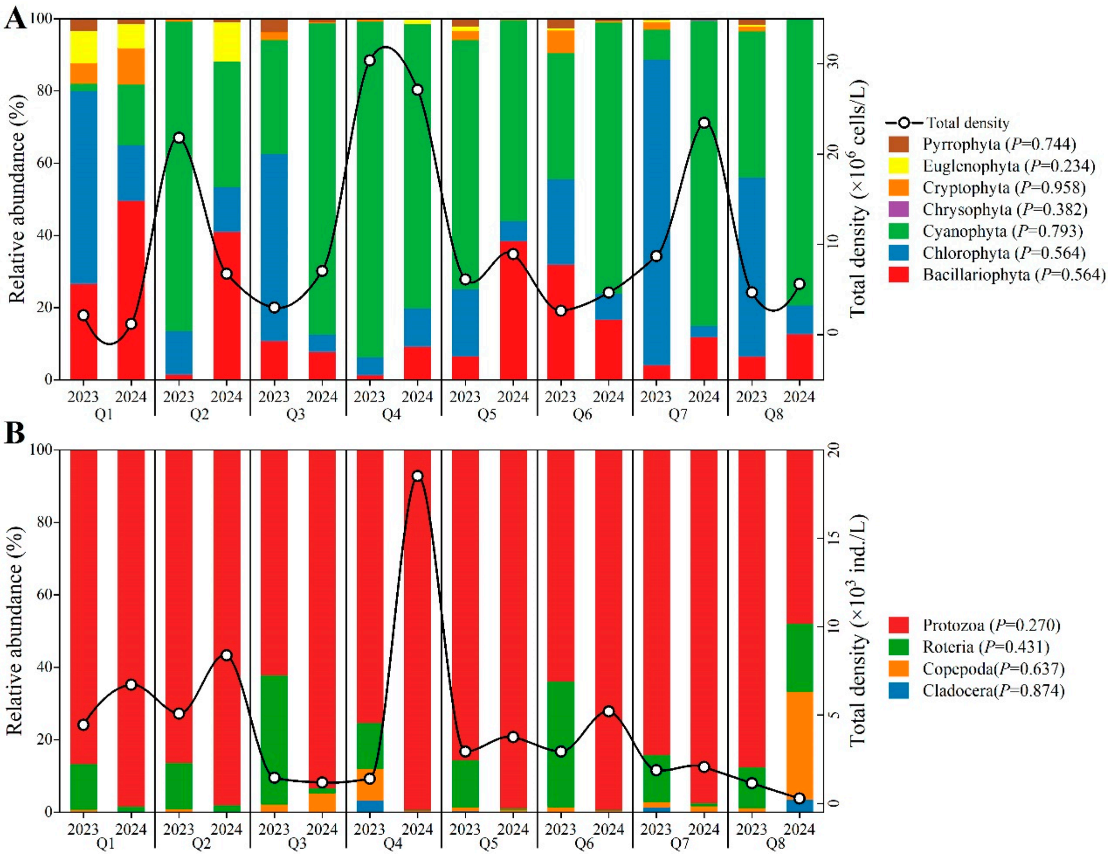

Figure S2. Variation in the relative abundance of plankton community composition in different sampling sites (A. Phytoplankton, B. Zooplankton).
